# Camera-type eye specific visual ontogeny in squid (*Sepioteuthis lessoniana*)

**DOI:** 10.1242/jeb.250437

**Published:** 2026-04-29

**Authors:** Chikatoshi Sugimoto, Shuhan Lei, Toru Ao, Yuma Sakurai, Xiumei Zhang, Yuzuru Ikeda

**Affiliations:** ^1^ Department of Chemistry, Biology and Marine Sciences, Graduate School of Science and Engineering, University of the Ryukyus, Okinawa 903-0213, Japan; ^2^ Department of Chemistry, Biology and Marine Sciences, Faculty of Science, University of the Ryukyus, Okinawa 903-0213, Japan; ^3^ The Key Laboratory of Mariculture (Education Ministry of China), Ocean University of China, Qingdao 266003, China; ^4^ Medical College of Qingdao University, Qingdao 266071, China; ^5^ Kyushu-Showa Co., Ltd, Fukuoka 812-0878, Japan; ^6^ Department of Biological Sciences, Faculty of Science, Hokkaido University, Sapporo 060-0810, Japan; ^7^ College of Fisheries, Zhejiang Ocean University, Zhejiang 316022, China

**Keywords:** Cephalopod, Visual acuity, Visual field, Anatomy, Behavior

## Abstract

The well-developed vision of vertebrates possessing camera-type eyes is rapidly established during the early phases of life. Cephalopods, a group of mollusks, possess well-developed camera-type eyes similar to those of vertebrates. Although the anatomy of the cephalopod visual system has been studied as an example of convergent evolution between vertebrates and invertebrates, knowledge of their vision and ontogeny is limited. Therefore, this study focused on the squid *Sepioteuthis lessoniana* to anatomically and behaviorally trace the ontogeny of vision, which includes eye anatomy, visual acuity and visual field during the 2 months post-hatching, to examine whether there are commonalities in the ontogenic features of camera-type eyes. Visual acuity estimated behaviorally rapidly increased during the first 2 weeks post-hatching, whereas visual acuity estimated anatomically increased continuously during the post-hatching phase and was accomplished by increasing the lens diameter and decreasing the density of visual cells with the thickening of the rhabdomeric layer. In contrast to the difference of the ontogenic features of visual acuity between behavioral and anatomical examination, the visual field exhibited a remarkable increase during the first week post-hatching in both examinations and this occurred along with a change in the orientation of the eyeballs in the head region. These results highlight that the common ontogenic feature of cephalopod vision is comparable to that of vertebrate vision, and indicates a specific early survival strategy in cephalopods with a short life span and no overlapping generations.

## Teaser

**Summary:** The rapid development of visual acuity and the visual field during the early growth phase in the cephalopod is a common ontogenic feature of camera-type eye specific vision.

## INTRODUCTION

Animal eyes are anatomically distinguished into compound and chambered eyes (Fernald, 2006), and vision is generally evaluated based on visual acuity (i.e. preciseness) and visual field (i.e. wideness). Compound eyes focus on a wider visual field rather than higher visual acuity (Pichaud and Casares, 2022), whereas chambered eyes, such as camera-type eyes, produce higher visual acuity with a large lens and a focus control system (Caves et al., 2018; Land, 2012). Generally, eyes develop ontogenically along with growth (increase in body size), wherein visual acuity and visual field noticeably develop (visual acuity, humans: Leat et al., 2009; monkeys: Teller et al., 1978; cats: Mitchell et al., 1976; birds: Schmid and Wildsoet, 1998; fishes: Shand, 1997; visual field, humans: Lewis and Maurer, 1992; cats and monkeys: Sireteanu, 1996; fishes: Collin and Shand, 2003). The development of visual acuity and the visual field is commonly initiated rapidly during the early life stages in these vertebrates. This fact raises the hypothesis that the rapid development of vision encompassing visual acuity and the visual field in camera-type eyes would be achieved with respect to survival strategies, such as parental care (e.g. mammals and birds) and metamorphosis (e.g. fishes), which are common phenomena soon after birth. This is because parental care compensates for the vulnerability of juveniles arising from their immature vision, and metamorphosis is related to the development of behavior and vision during habitat shifts (Beaudet and Hawryshyn, 1999).

Although camera-type eyes are dominant in vertebrates, they do exist in a few invertebrates. Coleoid cephalopods (octopus, cuttlefish and squid), a group of mollusks, are an example (Nilsson et al., 2012; Partridge, 2012; Saibil, 1990). Similar to those of vertebrates, the camera-type eyes of cephalopods are prominent in size, proportionate to body size, and exhibit high visual acuity (Caves et al., 2018; Makino and Miyazaki, 2010; Young, 1962). A common hypothesis is that cephalopods have evolutionarily acquired this vision because of competition among rival vertebrates such as fishes (Napoli et al., 2022; Packard, 1972). Cephalopods have sophisticated visual systems and large brains that are equivalent in size to those of vertebrates (Packard, 1972). However, large differences between cephalopods and vertebrates are also observed in their visual systems. Cephalopods show visually guided complex behaviors such as advanced learning, as in vertebrates; however, their life span is short (usually around 1 year), and parental care is absent because adults die soon after spawning (squid and cuttlefish) or soon after egg care until hatching (octopus). Moreover, cephalopods do not exhibit metamorphosis but instead direct development after birth. In particular, paralarvae of squid and octopus (the first post-hatching growth stage) possess a body plan resembling a miniature version of the adult and typically exhibit a planktonic lifestyle which is an obviously different characteristic from older individuals (Young and Harman, 1988). Furthermore, cephalopod eyes are embryologically of ectodermal origin and anatomically possess a non-converted retina. Vertebrates possess a fovea or area centralis, a specific pit or indentation structure on the retina with a high density of retinal cells, which provides high visual resolution and binocular vision (Collin and Shand, 2003; Jones et al., 2007). In contrast, cephalopods (except in one deep-sea species; Chung and Marshall, 2017) lack a fovea per se, but a streak or centralized area is observed in octopus, cuttlefish and squid (Talbot and Marshall, 2011). These similarities and differences present cephalopods as an optimal model for investigation of the ontogeny of vision for comparison with that of vertebrates. If the development of visual acuity and the visual field also begins rapidly during early life stages in cephalopods, this suggests that this characteristic may be a common feature of camera-type eyes.

Although the anatomy of cephalopod eyes has been extensively documented (e.g. extraocular muscle: Budelmann and Young, 1993; iris: Froesch, 1973; pupil: Talbot and Marshall, 2011; lens: West et al., 1995; retina: Yamamoto et al., 1965; retinal cells: Young, 1962; visual pigments: Brown and Brown, 1958), knowledge on vision and its ontogeny is quite limited, with the exception of a few studies (Bozzano et al., 2009; Groeger et al., 2005; Hao et al., 2010; Packard, 1969). Considering this background, we traced the ontogenic process of retinal anatomy, visual acuity and the visual field of the oval squid, *Sepioteuthis lessoniana*, using behavioral and anatomial experiments to test our hypothesis.

## MATERIALS AND METHODS

### Squid collection and rearing

The oval squid (*Sepioteuthis lessoniana* Férussac in Lesson 1932) inhabits the subtropical and tropical waters of the Indian and Pacific oceans (Jereb and Roper, 2006). This squid includes a species complex that can be divided into three reproductively isolated taxa: Aka-ika (red squid), Shiro-ika (white squid) or Aori-ika (common name in Japanese), and Kua-ika (quacking or small squid). Their habitats overlap in the coastal waters of the Ryukyu Archipelago in Japan (Segawa et al., 1993a,b). *Sepioteuthis* spp. have been identified via molecular analysis as different species (Izuka et al., 1994, 1996) and are currently referred to as *Sepioteuthis* sp. 1 (Aka-ika), *Sepioteuthis* sp. 2 (Shiro-ika) and *Sepioteuthis* sp. 3 (Kua-ika) (Imai and Aoki, 2012). Shiro-ika is genetically identical to *S. lessoniana*, which is distributed throughout mainland Japan (Imai and Aoki, 2012). Hereafter, we use the term *S. lessoniana* to refer to both *S. lessoniana* and *Sepioteuthis* sp. 2.

Egg masses of *S. lessoniana* for behavioral examination of visual acuity and the visual field and for anatomical examination of the visual field were collected from the southern area of Kin Bay, Okinawa Island, Ryukyu Archipelago, Japan, and specimens for anatomical examination of visual acuity were collected from the southeast area of Nago Bay, Okinawa Island, Ryukyu Archipelago, Japan. They were then transported to Ikeda Laboratory in the Department of Chemistry, Biology and Marine Sciences at the University of the Ryukyus, Nishihara Campus. Eggs and hatchlings were raised in a cylindrical tank connected with a closed-system (volume, 20 l; height, 350 mm; diameter, 300 mm; Multi-hydense^®^, Aqua Co., Ltd, Tokyo, Japan). We defined the day on which the most hatching occurred as day 0. The squid were reared until 63 days of age. Rearing conditions, such as the environment and feeding, followed protocols described by Sugimoto and Ikeda (2012, 2013).

### Ethical statement

Experiments with live cephalopods are not regulated by the Japanese government and are therefore not regulated at the University of the Ryukyus, where all experiments were conducted. However, we reared *S. lessoniana* following the rules generally applied in countries within the European Union (Smith et al., 2013).

Live juvenile guppies (*Poecilia reticulata*) were used for behavioral experiments. Because the University of the Ryukyus does not regulate the use of teleost fish for experiments, guppies were not used under specific ethical guidelines; however, we did not stress the fish further, except for their use as a live food source for squids.

### Behavioral examination for determination of visual acuity

Among the typical behavioral methods used to estimate visual acuity, i.e. using optomotor responses or training to discriminate, and measuring reactive distance (Groeger et al., 2005), we employed measurement of reactive distance. This was the easiest and most suitable measurement for squid, which are difficult to control in captive conditions, and therefore detecting valid responses by the traditional method using optomotor responses becomes challenging. To trace the ontogenic process of squid vision, discrimination training would not be applicable because of the small size of the juveniles. Observing the reactive distance in hunting behavior is more appropriate because the behavior emerges soon after hatching in cephalopods (Boletzky, 1974; Chen et al., 1996; Villanueva et al., 1996). Typical features of hunting behavior in squid, namely stalking and pursuit (Hanlon and Messenger, 2018), help with detection of reactions because of their large range of motion compared with ambushing (sit and wait) in benthic octopuses and cuttlefish (Hanlon and Messenger, 2018; Sugimoto and Ikeda, 2013).

**Fig. 1.**
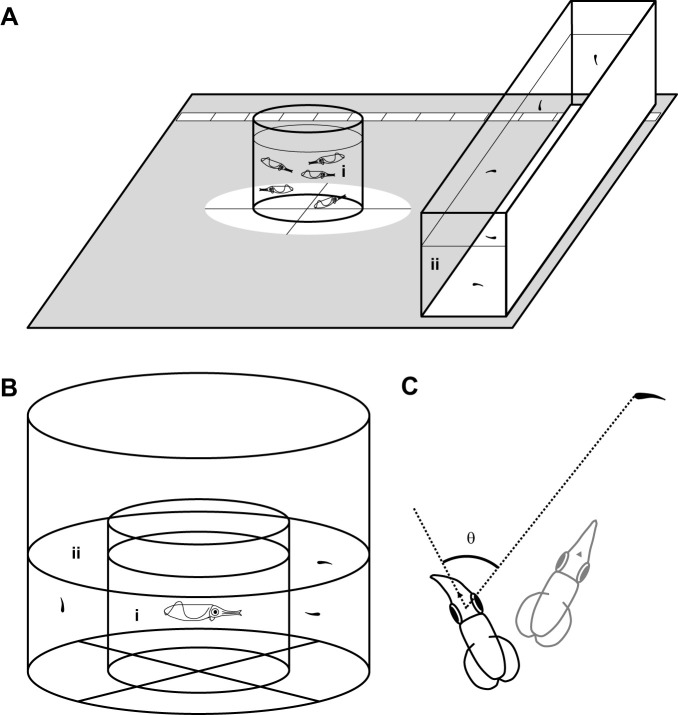
**Experimental apparatus for measuring vision by behavioral examination.** (A) Visual acuity was estimated based on the distance between the squid and the target (guppy fry) just one frame (30 frames s^−1^) before the squid oriented toward the target. The subject space (height, 170 mm; diameter, 160 mm) (i) containing 5 squid (3 squid at 0 days old; dorsal mantle length ranged from 6.21 mm for 0 days of age to 40.23 mm for 63 days of age) was moved away from the closest position to the target space (l×w×h, 160×510×200 mm) (ii) containing 5 guppy fry (the total length was 7.42±1.89 mm, mean±s.d.). (B) Visual field was estimated based on the angle between the head axis of a squid and the target (guppy fry) just one frame (30 frames s^−1^) before the squid oriented toward the target. The subject space (height, 170 mm; diameter, 160 mm) (i) containing one squid (dorsal mantle length ranged from 5.92±0.27 mm for 0 days of age to 35.75±4.68 mm for 63 days of age, mean±s.d.) was centered in the target space (height, 300 mm; diameter, 300 mm) (ii) containing 3 guppy fry (the total length was 7.42±1.89 mm, mean±s.d.). A high-vision digital video camera was set above both experimental apparatus to simultaneously record the behavior of the squid and guppy fry. (C) Target angle (θ), the angle between the head axis and a line to the target.

To estimate visual acuity by observing the hunting behavior of the squid, an experimental apparatus consisting of a transparent cylindrical enclosure (height, 170 mm; diameter, 160 mm) as the space for the subject (the squid) and a transparent rectangular tank (l×w×h, 160×510×200 mm) as the space for the target animal (the guppy fry) was used (Fig. 1A). Because this apparatus was located on a pedestal submerged in a square fiber-reinforced plastic (FRP) tank (l×w×h, 1800×1800×600 mm; volume, 1500 l; Aqua Co., Ltd), the water depth was adjusted to 140 mm. The distance between the subject and target spaces (between the center points of the subject and target spaces) was set at 240–1320 mm. A high-vision digital video camera (ivis HFS11, Canon Inc., Tokyo, Japan) was set above the experimental apparatus to simultaneously record (30 frames s^−1^) the behavior of the squid and guppy fry. To enhance the contrast between the animals, a white plastic sheet was placed under both the subject and target spaces. A white sheet was placed on the outer wall of the target space to enhance the visibility of the guppy fry. Light was provided naturally through windows and artificially through fluorescent lamps above the experimental apparatus. The light intensity was measured every 3 days during the experimental period at 5–10 locations surrounding the subject and target space in the water using a luminometer (T-10Ws, Konica Minolta Inc., Tokyo, Japan) within a range of 170 to 230 lx. This light intensity and non-uniform lighting correspond to the light conditions used in a standard rearing environment for the squid (Sugimoto and Ikeda, 2012, 2013). Despite the light being significantly lower in intensity than in their natural habitats in shallow waters, sufficient hunting behavior necessary for natural growth and data collection was observed (Sugimoto and Ikeda, 2012, 2013).

Hunting behavior of the squid was observed once per week from 0 to 63 days of age. Observations were conducted when the squid were hungry (at least 2 h after the previous feeding). After the introduction of 5 squid (except for 3 squid at 0 days old) randomly selected from the rearing tank into the subject space, a habituation period (5–10 min) was allowed before the onset of video recording. Five guppy fry were introduced into the target space 20 s after the onset of the video recording. We recorded the position of the guppy fry and the hunting behavior of the squid for 3–5 min. We judged the following motion as hunting behavior: after finding a target, the squid kept chasing the target and pushing the tank wall of the subject space with its arms. At the beginning of each experimental day, to confirm the hunting motivation of the squid, the subject space was placed closest to the target space (at 240 mm) (Table 1). After finishing a recording, to distinguish experimented individuals, the squid in the subject space were temporally transferred to a spare tank until all recordings for the day were completed. Recordings were conducted twice at each position in the subject space, i.e. 10 squid in total (except for six squid at 0 days of age). After recording at one position, the subject space was moved farther away from the target space (an additional 100 mm was added each time to the initial distance of 240 mm) until the squid exhibited no reaction. To effectively determine the position showing no reaction (i.e. invisibility) by the squid, after the first recording at 240 mm, the subject space was moved to the position where the squid had exhibited no reaction on the previous experimental day. The experimented squid placed in the temporary spare tank were returned to the rearing tank after completing all the recordings for that experimental day.

**Table 1.** Number of oval squid that showed hunting behavior against the target animal (guppy fry)

Age (days)	Distance between subject and target spaces (mm)										
	240	340	470	550	680	770	880	1000	1110	1270	1320
0	5	−	2	0*	0	−	−	−	−	−	−
7	10	5	4	2	1	0	−	−	−	−	−
14	9	−	−	8	−	3	0	−	−	−	−
22	10	−	−	−	−	−	5	2	1	−	−
28	10	−	−	−	−	−	3	0	0	−	0
35	9	−	−	−	−	−	1	0	0	−	−
42	10	−	−	−	−	−	1	0	1	−	−
49	8	−	−	−	−	−	0	5	1	−	−
57	10	−	−	−	−	−	−	0	2	1	−
63	5	−	−	−	−	−	0	0	1	−	−

Recording was conducted for a total of 10 individuals for each distance at each age (one recording contained 5 individuals) except for 0 days of age, when a total of 6 individuals was used (one recording contained 3 individuals). Dashes indicate that no recording was conducted. *Recording was conducted once.

### Estimation of visual acuity from behavioral recordings

We counted the number of squid that showed hunting behavior in the subject space by visual observation of the experimental apparatus from a distance so as not to disturb the experimental animals. When no squid showed a response, the experiment ended. To estimate the maximum visual acuity on each experimental day, we used data from focal squid that showed hunting behavior at the farthest position of the subject space to the target prey. To identify the specific guppy targeted by the focal squid, recorded videos were played back after the onset of head movement to determine which prey attracted the squid's interest, then rewound to the beginning. Five guppy fry were present in the target space, but because the volume of the target space was sufficiently large relative to the size of guppy fry, they were typically scattered both horizontally and vertically. Furthermore, based on empirical evidence, because squid are thought to be sensitive to stimuli from the horizontal direction (squid should show a preference for guppy fry swimming at the same depth) and tended to persistently pursue the same target as at the onset of the hunting behavior, we successfully identified the targeted guppy. Using the recorded video images (19.37±2.25 pixels per inch, mean±s.d.), we manually measured the distance between the snout of a targeted guppy fry and the midpoint of the two eyes of the focal squid just one frame (30 frames s^−1^) before the point at which the squid's head axis began to change orientation toward the guppy fry at an arbitrary angle (target distance, TD). Ideally, we should analyze eye movements to detect the attention squid direct toward the targeted guppy. However, the resolution of the recorded video data was insufficient to track eye movements during a single hunting behavior. Additionally, the darker body coloration of the squid (cephalopods frequently change their body color) also hindered the tracking of eye movements. We also measured the total length of guppy fry (target length, TL; 7.42±1.89 mm, mean±s.d.) and the dorsal mantle length (ML) of the focal squid. These three values were measured using the software ImageJ^®^ (version 1.46, US National Institutes of Health, Bethesda, MD, USA; https://imagej.net/ij/). Visual acuity (VA) was calculated using the following formula (Hajar et al., 2008; Wanzenböck and Schiemer, 1989; Watanuki et al., 2000):
(1)

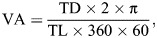
where TD×2π provides the circumference at radius TD, TL×360 is the conversion of TL circumference into degrees; and ×60 is the conversion of degrees to minutes (one-sixtieth of one degree is one minute). Visual acuity 1.0 is calculated as the inverse of the visual angle at 1 min (visual acuity 0.5 corresponds to a visual angle at 2 min, etc.). Because the visual angle is formed by the center of the subject's lens and both tips of the target, i.e. the TL (Wanzenböck and Schiemer, 1989), the size of the target can be expressed in 1 min under a visual acuity of 1.0. Visual acuity is expressed as cycles per degree (cpd). When we use black and white stripes as the target, under a visual acuity of 1.0, because the width of one band corresponds to 1 min, 1 deg contains 30 cycles (one cycle contains one set of neighboring black and white bands). Thus, a visual acuity of 1.0 corresponds to 30 cpd. The unit of visual acuity can be converted into cpd by multiplying by 30. In order to consider the relationship between the response of squid and the distribution of guppy fry, we obtained coordinate data of 5 guppy fry (by focusing on the snout) on the recorded video just one frame (30 frames s^−1^) before the focal squid initiated hunting behavior and calculated the interindividual distances for all pairs of guppy fry. The coordinate data were obtained using the software ImageJ^®^ (version 1.46, US National Institutes of Health; https://imagej.net/ij/) for the hunting behaviors that we used for calculation of visual acuity.

### Behavioral examination for determination of visual field

To behaviorally estimate the visual field, we used reactive distance during hunting behavior by the squid, which was also applied to determine visual acuity (Fig. 1B). In this situation, the squid could clearly see the target guppy fry. Although the visual field expands both horizontally and vertically, we focused on the horizontal visual field for technical reasons.

To estimate the visual field by observing the hunting behavior of the squid, an experimental apparatus consisting of a transparent cylindrical enclosure (subject space: height, 170 mm; diameter, 160 mm) inside another transparent cylindrical enclosure (target space: height, 300 mm; diameter, 300 mm) was used (Fig. 1B). Because this apparatus was located on a pedestal inside a square FRP tank, similar to the experimental setup for visual acuity, the water depth was adjusted to 140 mm. We placed a white plastic sheet under the experimental apparatus to enhance the contrast of the animals, and recordings were taken using the same device and light conditions as in the visual acuity experiment. A visual acuity examination was performed prior to the visual field examination to confirm that the squid could see the target animal under the experimental setup. The recording schedule for the visual field was the same as that for the visual acuity experiment. After returning all experimental squid for visual acuity to the rearing tank, one squid was randomly selected from the tank and introduced into the subject space. A habituation period (5–10 min) was allowed before the onset of video recording. Three guppy fry were introduced into the target space 20 s after the onset of the video recording. We recorded the position of the guppy fry and the hunting behavior of the squid for 3 min. After completing a recording, the squid in the subject space was temporarily transferred to a spare tank until all recordings for the day were completed. On each experimental day, 5 squid were observed for the visual field and then used for further examination as described in ‘Anatomical examination for determination of the visual field’, below.

### Estimation of visual field from behavioral recordings

The judgment criterion for the hunting behavior of squid was the same as that for visual acuity. From the recorded video images (101.78±12.32 pixels per inch, mean±s.d.), we manually measured the angle between the line from the midpoint of the two eyes of the squid to the snout of the target guppy fry, and a line from the midpoint of the eyes to the beak of the squid (head axis). We measured these angles immediately one frame (30 frames s^−1^) before the point at which the squid's head axis began to change orientation toward the guppy fry at an arbitrary angle (target angle, TA; Fig. 1C). The TA was measured for all hunting behaviors observed, and angle data were separately recorded for the left or right rotational direction for each of the 5 squid; that is, each TA was less than 180 deg. For each squid, the maximum TA was doubled to estimate its visual field. The ML was measured from the recorded video images of all squid used in the experiment. We used the software ImageJ^®^ (version 1.46, US National Institutes of Health; https://imagej.net/ij/) for measurement of TA and ML. In order to consider the relationship between the response of squid and the distribution of guppy fry, we obtained coordinate data of 3 guppy fry (by focusing on the snout) on the recorded video just one frame (30 frames s^−1^) before the focal squid initiated hunting behavior and calculated the interindividual distances for all pairs of guppy fry. The coordinate data were obtained using the software ImageJ^®^ (version 1.46, US National Institutes of Health; https://imagej.net/ij/) for the hunting behaviors that we used for calculation of the visual field.

### Anatomical examination for determination of visual acuity

To estimate visual acuity, the morphometric characteristics of the squid eye were observed. The specimens were randomly selected from the rearing tank. After anesthesia with 1–2% ethanol in seawater, the wet body mass and dorsal ML of the squid were measured using a digital balancer and a caliper. Immediately after the measurements, each squid was euthanized with 3–5% ethanol in seawater and the whole head was fixed in 10% formalin in seawater. The left and right eyeballs were removed from the head of each specimen (six squid at 0, 6, 13 and 20 days of age and 3 squid at 27, 34, 41, 48, 55 and 62 days of age). The retina was then removed, and an incision was made on the anterior side to confirm the direction of the bowl-shaped eyeball. Under a dissecting microscope, each retina was divided into several regions according to size. In the case of the small eyeball of younger squid, the retina was not cut into pieces (Table 2). The positions of these retinal pieces were mapped back to the contour of the retina, which was traced from a photograph of the retina taken just before cutting. Each retinal section was dehydrated using a graded series of ethanol, made transparent with xylene, and embedded in paraffin. Serial sections (5 µm) of the retina were prepared and stained with hematoxylin and eosin. Retinal sections from the left eye were cut in the radial direction, whereas those from the right eye were cut in the tangential direction to observe the retinal cells in different directions.

**Table 2.** Morphometric data for lenticular and retinal parameters detected by anatomical examination of the oval squid

Age (days)	No. of specimens	Dorsal mantle length (mm)	Wet body mass (g)	Lens diameter (mm)	No. of retinal pieces	Maximum density of visual cells (×10^2^ cells mm^−2^)	Diameter of visual cell nuclei (μm)	Maximum height of rhabdomeric layer (μm)
0	6	5.20±0.22	0.03±0.01	0.40±0.00	1	324–360	3.08±0.42	51.50±3.51
6	6	5.44±0.26	0.03±0.01	0.52±0.07	1	332−363	3.77±0.42	73.83±10.81
13	6	8.28±0.36	0.10±0.02	0.86±0.04	1	267−318	4.73±0.39	96.33±11.11
20	6	10.78±1.03	0.18±0.03	1.10±0.07	1	266−339	4.33±0.23	107.50±4.64
27	3	11.31±1.65	0.24±0.08	1.15±0.13	1	246−253	4.47±0.25	119.67±14.36
34	3	16.31±2.00	0.57±0.15	1.53±0.12	12	275*	4.60±0.17	126.00±7.00
41	3	18.13±2.06	0.72±0.24	1.63±0.21	12−18	301*	4.60±0.10	127.67±8.14
48	3	19.13±2.83	1.14±0.54	1.93±0.25	12−18	305*	4.53±0.40	144.67±7.09
55	3	27.90±2.12	2.24±0.40	2.50±0.35	18−24	248*	4.77±0.12	177.00±10.54
62	3	38.40±6.51	5.54±2.26	3.13±0.40	30−36	263*	4.43±0.12	183.00±8.19

Data are means±s.d. or range (asterisks indicate data from only one specimen).

### Morphometric measurement of the eye and visual acuity estimation

The diameter of the lens was measured during the dissection of the squid eyeball under a dissecting microscope. Retinal sections were observed using a light microscope (AXIO Imager, Carl Zeiss Industrielle Messtechnik GmbH, Oberkochen, Germany) connected with a computer (iMac^®^, Apple Inc., Cupertino, CA, USA) for taking photographs. The maximum height of the rhabdomeric layer and diameter of the retinal cell nuclei were measured using the photomicrographs with the software ImageJ^®^ (version 1.46, US National Institutes of Health; https://imagej.net/ij/). The maximum height of the rhabdomeric layer was measured as the highest value of the rhabdomeric layer among the retinal pieces in the left eye for each specimen. The highest point of the rhabdomeric layer in one retinal piece was decided through comparison among the heights of several points of the layer. The diameter of the retinal cell nuclei was measured from cells in the deeper layer of the inner segment of the retinal cells, as described by Makino and Miyazaki (2010) and averaged among 10 randomly selected cells from the retinal pieces of the right eye for each specimen. In addition, the number of retinal cell nuclei in a 0.01 mm^2^ section was counted in each retinal piece. The number of retinal cell nuclei was counted twice to ensure data reliability. If the count differed by more than 5%, we selected a new region in the same photomicrograph for counting. We built an iso-density contour map of retinal cell nuclei using the counted number on each retinal piece sampled from each retina by connecting the isopycnic points with a smooth curve. Visual acuity (VA) was calculated using the following formula (Hajar et al., 2008; Tamura, 1957; Torisawa et al., 2007):
(2)

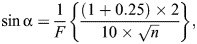

(3)

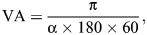
where α is the minimum separable angle in radians, *F* is the focal length of the lens calculated as 2.55 times the radius of the lens in mm (the Matthiessen's ratio), 0.25 is the degree of shrinkage by formalin fixation, *n* is the maximum number of retinal cell nuclei per 0.01 mm^2^, 10×√*n* is the number of retinal cell nuclei along a 1 mm line and 2 is the number of gaps among the three retinal cells comprising the minimum number of cells required to distinguish one target from the background; 180/π is the conversion of radians to degrees; and 60 is the conversion of degrees to minutes (one-sixtieth of one degree is one minute). Visual acuity is expressed as cpd.

### Anatomical examination for determination of visual field

**Fig. 2.**
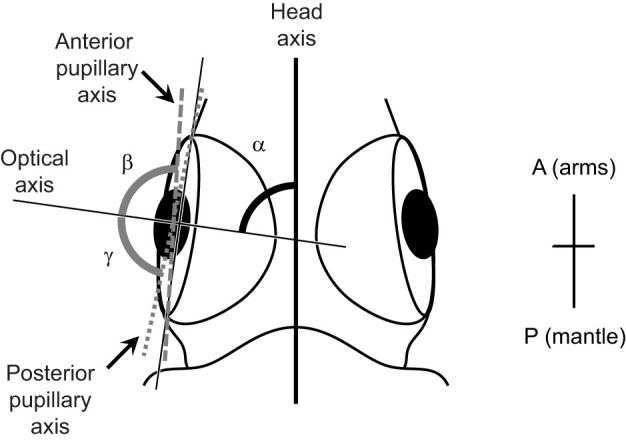
**Definition of visual field in the oval squid by anatomical examination.** The optical axis angle (α) is the angle between the head axis and optical axis. The anterior uniocular angle (β) is the angle between the optical and anterior pupillary axes. The posterior uniocular angle (γ) is the angle between the optical and posterior pupillary axes. A, anterior; P, posterior.

Similar to previous studies on cuttlefishes (Watanuki et al., 2000; Messenger, 1968), the horizontal extension of the visual field of the oval squid was anatomically estimated based on the positions of the anterior and posterior edges of both the pupil and retina, i.e. eye orientation, which were derived from the position of the eyes for squid under anesthesia (Fig. 2). Specimens were collected from among individuals used in the experiment described above in ‘Behavioral examination for determination of visual field’. After anesthesia with 1–2% ethanol in seawater, 5 squid observed on a single day of the behavioral experiment were photographed from the dorsal side using a digital camera (EOS Kiss X3, Canon Inc.).

### Estimation of visual field from eye orientation

Based on the obtained images, the head, optical, anterior pupillary and posterior pupillary axes were defined as described in Fig. 2. The head axis is the line from the midpoint between the eyes and beak. The optical axis is a vertical line from the anterior to the posterior edge of the lens plane, i.e. the anterior and posterior ends of the retina, through the midpoint of the lens. The anterior pupillary axis is a line from the posterior end of the retina to the anterior edge of the pupil, and vice versa for the posterior pupillary axis. The optical axis angle was measured as the angle between the head and optical axes. The anterior uniocular angle was measured as the angle between the optical and anterior pupillary axes, and the posterior uniocular angle was measured as the angle between the optical and posterior pupillary axes. The uniocular visual field was defined as the sum of the anterior and posterior uniocular angles. The binocular visual field was defined as the overlap of the uniocular visual field of each eye anteriorly and posteriorly. The visual field was defined as the sum of the uniocular visual fields of the left and right eyes minus the binocular visual field of the anterior and posterior axes. The ML was also measured from the photographs of all the squid used in the measurement. We used the software ImageJ^®^ (version 1.46, US National Institutes of Health; https://imagej.net/ij/) for these measurements.

### Disparity in the estimation of visual acuity depending on calculation methods

In previous cephalopod studies investigating visual acuity using behavioral examination, despite methodological variation, the calculation method for visual acuity was the same. That is, there are two variables: the size of the target and the distance between the target and subject animals. In contrast, in anatomical studies, two variables, lens size and the distance between each retinal cell, are used to estimate visual acuity, using two main calculation methods. Because a large difference in the consequent visual acuity emerged between these calculation methods, we must take care when comparing the results of previous studies. One method is to estimate visual acuity as the minimum separatable angle, which we employed in our study, as previously described (Hajar et al., 2008; Tamura, 1957; Torisawa et al., 2007; Watanuki et al., 2000). An alternative method is to estimate visual acuity as cpd (Collin and Pettigrew, 1989; Makino and Miyazaki, 2010). The biggest difference between these two methods is the number of retinal cells required to detect a target. Method 1 fundamentally requires three retinal cells to detect both boundaries of a single target, such as prey animals and the gap of the Landolt-C mark; method 2 requires two cells to detect one boundary between the black and white stripes. This difference produces a relatively wide variance in the estimated values of visual acuity. Correction for tissue shrinkage by formalin fixation in method 1 also enlarged this difference. This results in a misunderstanding of the results by direct comparison of values from anatomical studies, even if the unit is the same. However, because the visual field was anatomically estimated based on the position of the pupil (Messenger, 1968; Watanuki et al., 2000), the influence of methodological variation was probably not high.

### Statistical methods

Correlation analyses were conducted using the software KaleidaGraph (version 4.0, Synergy Software, Reading, PA, USA; https://www.synergy.com). To clearly track the temporal changes in data from behavioral and anatomical experiments for visual acuity and the visual field, an appropriate regression line was selected from linear, logarithmic or exponential functions according to the R-value. The relationship between the response of squids and the distribution of guppy fry was analyzed using generalized linear models (GLMs) using the lme4 package in R (version 4.4.2, R Core Team 2024, Vienna, Austria; https://www.R-project.org) for behavioral experiments for measurement of visual acuity and the visual field.

## RESULTS

### Ontogenic changes in behaviorally estimated visual acuity

Recording for visual acuity was conducted for a total 21 individuals for 0 days of age, 60 individuals for 7 days of age, 50 individuals for 28 days of age and 40 individuals for all other ages (Table 1). Throughout the experimental period, a high motivation for hunting was shown by squid that reacted to guppy fry at the position closest to the subject space (i.e. 240 mm; except for 63 day old squid, wherein only half of the squid reacted). Two to three 0, 14 and 28 day old squid exhibited hunting behavior at the farthest position on each experimental day. In contrast, for all other ages, only a single squid exhibited hunting behavior.

**Fig. 3.**
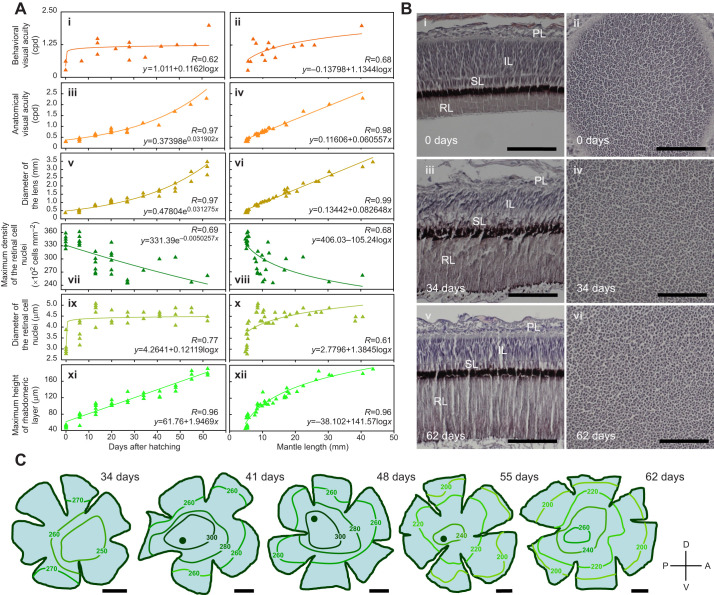
**Ontogeny of visual acuity in the oval squid.** (A) Ontogeny of visual acuity values in relation to age (left) and mantle length (right) in the oval squid; visual acuity determined by behavioral examination (i,ii) or anatomical and histological examination (iii,iv), lens diameter (v,vi), maximum density of the retinal cell nuclei (vii,viii), diameter of the retinal cell nuclei (ix,x) and maximum height of the rhabdomeric layer (xi,xii). A linear regression (*y*=294.18−0.77563*x*; *R*=0.245) for maximum density of the retinal cell nuclei (viii) was derived from data of individuals older than 14 days. (B) Ontogeny of the retina in the radial (left) and tangential (right) section in the oval squid. Scale bars: 0.1 mm. IL, inner segmental layer; PL, plexiform layer; RL, rhabdomeric layer; SL, subrhabdomeric layer. Anatomical definition following Hao et al. (2010), Yamamoto et al. (1965) and Young (1962). The number of days after hatching is indicated. (C) Iso-density (×10^2^ cells mm^−2^) contour maps of retinal cell nuclei in the oval squid. Scale bars: 1 mm. The black circle indicates the position of the highest cell density. The number of days after hatching is indicated. A, anterior; P, posterior; D, dorsal; V, ventral.

Based on the behavioral experiment, visual acuity of the squid logarithmically increased with age (Fig. 3Ai) and growth (Fig. 3Aii), showing rapid development during the first 2 weeks post-hatching. The visual acuity was estimated as 0.63 cpd (6.21 mm ML) for 0 day old, 0.65 cpd (5.48 mm ML) for 7 day old, 1.50 cpd (7.01 mm ML) for 14 day old, 1.11 cpd (11.26 mm ML) for 22 day old, 1.35 cpd (11.73 mm ML) for 28 day old, 0.78 cpd (13.79 mm ML) for 35 day old, 1.20 cpd (19.43 mm ML) for 42 day old, 1.26 cpd (21.97 mm ML) for 49 day old, 1.26 cpd (25.78 mm ML) for 57 day old and 2.01 cpd (40.23 mm ML) for 63 day old squid (Fig. 3Ai,ii). Only the maximum values are shown from the two to three values obtained at 0, 14 and 28 days of age.

Croft et al. (2006) used a criterion (within four body lengths) for the interindividual distance of guppies within the same shoal. In our study, because only 11% of all interindividual distances recorded at the onset of the hunting behaviors that we used for the calculation of visual acuity showed a shorter interindividual distance than the criterion, the guppy fry basically did not shoal in our experimental apparatus. The distribution bias of left or right guppy fry (more than half of the 5 guppy fry were distributed or not) relative to a line extending the head axis at the onset of the hunting behaviors was 53% and 47%, respectively. A GLM with binomial error distribution and logit link function revealed no significant difference between them (estimate±s.e. −0.13±0.52, *z*=−0.26, *P*=0.80; predicted probability of left-side 0.53 [95% confidence interval, CI: 0.29−0.76], right-side 0.47 [95% CI: 0.24−0.71]).

### Ontogenic changes in anatomically estimated visual acuity

Anatomical examination for visual acuity was conducted for a total 6 specimens from 0 to 20 days of age and 3 specimens from 27 to 62 days of age (Table 2). Because some failures occurred during the process of sectioning the retina, we analyzed data for one squid at 34–62 days of age to determine the maximum density of retinal cell nuclei and visual acuity. To analyze the diameter of retinal cell nuclei, we assigned the average ML of each age to one of the 3 specimens at 41, 48, 55 and 62 days of age because of the loss of data.

The retina of the oval squid examined in this study comprised several layers. Therefore, we labeled these layers as the rhabdomeric (distal segments), subrhabdomeric, inner segmental (proximal segments) and plexiform (retinal nerve plexus) layers, according to previous studies (Hao et al., 2010; Yamamoto et al., 1965; Young, 1962) (Fig. 3Bi,iii,v). Retinal cell nuclei were localized in the inner segmental layer (Fig. 3Bii,iv,vi). The fundamental structure of the retina was almost the same at different ages, whereas the rhabdomeric layer rapidly thickened in height with age (Fig. 3 Bi,iii,v).

Morphometric data for lenticular and retinal parameters are summarized in Table 2. The diameter of the lens exponentially enlarged as the squid aged (Fig. 3Av) and linearly enlarged with growth (Fig. 3Avi). The maximum density of retinal cell nuclei showed a linear decrease as the squid aged (Fig. 3Avii) and exhibited a logarithmic decrease with growth (Fig. 3Aviii). In contrast, the diameter of the retinal cell nuclei rapidly increased until 14 days of age, remained constant thereafter (Fig. 3Aix), and logarithmically enlarged with growth (Fig. 3Ax).

Based on the anatomical experiment, visual acuity increased exponentially as squid aged (Fig. 3Aiii), and increased linearly with growth (Fig. 3Aiv). The visual acuity was estimated as 0.34 cpd (5.33 mm ML) for 0 day old, 0.50 cpd (5.85 mm ML) for 6 day old, 0.69 cpd (8.09 mm ML) for 13 day old, 0.95 cpd (12.02 mm ML) for 20 day old, 0.91 cpd (13.19 mm ML) for 27 day old, 1.18 cpd (16.70 mm ML) for 34 day old, 1.08 cpd (17.20 mm ML) for 41 day old, 1.71 cpd (22.40 mm ML) for 48 day old, 2.03 cpd (30.30 mm ML) for 55 day old and 2.31 cpd (40.50 mm ML) for 62 day old squid (Fig. 3Aiii,iv). Only the maximum values are shown at 0 to 27 days of age.

In evaluations of vision with parameters other than resolution as visual acuity, optical sensitivity has been estimated using parameters such as pupil size (aperture diameter), retinal cell (diameter and length of photoreceptor) and lens (focal length) (Bozzano et al., 2009; Talbot and Marshall, 2010; Warrant and Nilsson, 1998). Because retinal cells absorb light from the rhabdomere (Matsui et al., 1988; Seidou et al., 1990), the height of the rhabdomeric layer is a rough estimate of the sensitivity to light. The maximum height of the rhabdomeric layer linearly increased as squid aged (Fig. 3Axi) and logarithmically increased as squid grew in size (Fig. 3Axii), showing rapid development of light sensitivity during the early phase post-hatching.

Owing to some technical difficulties in observing the retinas of tiny eyes in squid younger than 1 month post-hatching, we could only draw whole retinal iso-density maps for 34 to 62 day old squids. Regarding the retinal cell nuclei, a noticeable difference in density between the peripheral and central areas of the retina was absent at 34 days of age; however, after 41 days of age, the highest density region appeared in the posterior and slightly dorsal or middle areas of the retina (Fig. 3C).

### Ontogenic changes in behaviorally estimated visual field

Recording for the visual filed was conducted for a total 50 individuals from 0 to 63 days of age (Table 3). Although all individuals from 7 to 57 days of age showed at least one hunting behavior, 2 individuals for 0 days of age and 1 individual for 63 days of age did not show any hunting behavior. The number of hunting behaviors exhibited by each individual ranged from 1 to 11 times, with 1 to 3 times accounting for over 60% of the total.

**Table 3.** Visual field of the oval squid detected by hunting behavior against the target animal (guppy fry)

Age (days)	Target angle (deg)																														
0	**5**	42	**15 |**	69	82	**60**	80	21	**27 |**	**40**	**52**																				
7	**103**	**146**	**5**	**10**	**23**	90	140	71	97	7	90 **|**	**28**	**124**	134	**144 |**	22 **|**	**113**	**78**	114	**81**	**72**	**14 |**	117	16	**26**	**150**	**3**	8	**8**	**51**	**54**
14	158	**171**	**139**	17	**14 |**	70	129	**82 |**	115	**45**	101	**127**	93	35	**4 |**	**142 |**	**139**	7	**45**												
22	**119**	**103**	117	7	15 **|**	**40 |**	166	**111**	**24**	**14**	**6**	**10 |**	124	**57**	**62**	66	**168 |**	138	16	**136**	**145**	**87**	127								
28	**66**	**11**	134	28	6	**16**	102 **|**	108	36 **|**	135	67 **|**	172	**51**	**20 |**	**63**	18	**66**	58													
35	**93**	**26**	**8**	152 **|**	**157**	**137 |**	142	30	48	35	53	**37 |**	88	**67 |**	51	156	10	**53**	**21**												
42	166 **|**	**126**	38	159 **|**	149	32	**6 |**	**115 |**	**52**	**4**	**6**	**151**	13	**37**	**30**	**162**															
49	**44**	**23 |**	60	**61 |**	165	155	**89**	**156 |**	**114 |**	73	**138**																				
57	**174 |**	**14**	61 **|**	**36**	29	**6 |**	22	**143**	**17 |**	93	**166**																				
63	**15**	**8 |**	**15 |**	**9**	39 **|**	**122**																									

Target angle was calculated as the angle between a line from the midpoint of the two eyes of the squid to the snout of the target guppy fry, and a line from the midpoint of the eyes to the beak of the squid (head axis) just before the squid oriented toward the target. Visual field was estimated by doubling the maximum target angle. Data list the orientation toward prey over the course of the 3 min video recording. Vertical bars separate data from different squid individuals. Note, two individuals of 0 days of age and one individual of 63 days of age did not show hunting behavior during the video recording. Numbers in bold indicate location of the guppy fry on the right and left sides of the head axis of the squid, respectively.

**Fig. 4.**
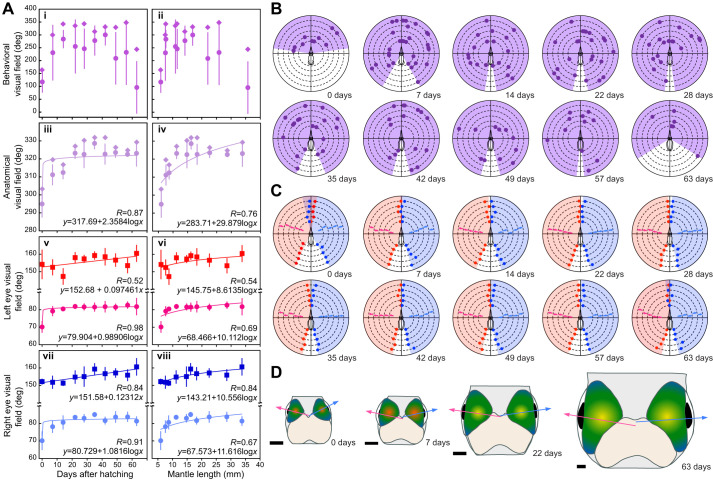
**Ontogeny of visual field in the oval squid.** (A) Ontogeny of the visual field values in relation to age (left) and mantle length (right) in the oval squid; visual field determined by behavioral examination (i,ii) or anatomical examination (iii,iv), optical axis angle (circles) and uniocular visual field (squares) in the left (v,vi) and right eye (vii,viii). Symbols (square/circles) and error bars indicate means and standard deviation, respectively. Diamonds indicate the maximum value on each experimental day. (B) Ontogeny of the visual field in the oval squid determined by behavioral examination. Circles on the dashed lines indicate the position at which a squid found a target (guppy fry). Data from 5 squid are shown as dashed lines; absence of a circle indicates that these individuals found no target for that experimental day. Maximum visual field is shown by the purple shading at each age. The number of days after hatching is indicated. (C) Ontogeny of the visual field in the oval squid determined by anatomical examination. Data from 5 squid are shown as dashed lines. Red and blue circles indicate left and right uniocular visual fields, respectively. Pink and light blue arrows indicate the left and right optical axis, respectively. The maximum value for each of the left and right uniocular visual fields is shown by the red and blue shading, respectively. The number of days after hatching is indicated. (D) Ontogeny of size and position for the eyes and brain (optic lobes and central nervous system) in the oval squid. Pink and light blue arrows indicate the left and right optical axis, respectively. Scale bars: 1 mm. The number of days after hatching is indicated.

Based on the behavioral experiment, the distribution of the target angle expanded as the squid aged (Fig. 4B and Table 3). Moreover, the visual and maximum visual fields became considerable wider during the first week post-hatching, and then remained constant (Fig. 4Ai,B). A similar trend was observed for growth in body size (Fig. 4Aii). The visual field was estimated as 116.94±41.96 deg, 164.09 deg (mean±s.d., maximum) (5.92±0.27 mm ML) for 0 day old, 230.51±108.18 deg, 300.09 deg (7.51±0.44 mm ML) for 7 day old, 283.46±35.31 deg, 342.68 deg (7.69±0.23 mm ML) for 14 day old, 254.86±105.03 deg, 335.73 deg (10.94±0.43 mm ML) for 22 day old, 245.84±78.23 deg, 343.35 deg (11.54±1.14 mm ML) for 28 day old, 277.72±58.37 deg, 313.27 deg (14.19±1.19 mm ML) for 35 day old, 299.88±41.45 deg, 331.31 deg (16.84±1.26 mm ML) for 42 day old, 208.59±101.86 deg, 329.53 deg (22.03±2.51 mm ML) for 49 day old, 231.76±126.50 deg, 347.920 deg (25.88±3.53 mm ML) for 57 day old and 95.43±101.97 deg, 244.39 deg (35.75±4.68 mm ML) for 63 day old squid (Fig. 4Ai,ii).

Because 3 individuals were mistakenly presented with two guppy fry during the entire recording period or immediately after its start, we excluded all or part of their data from the analysis about the relationship between the response of squid and the distribution of guppy fry. According to the criterion (within 4 body lengths) for the interindividual distance of guppies within the same shoal (Croft et al., 2006), because only 10% of all interindividual distances recorded at the onset of the hunting behaviors that we used for the calculation of visual field showed a shorter interindividual distance than the criterion, the guppy fry did not shoal in our experimental apparatus. The distribution bias of left or right guppy fry (more than half of the 3 guppy fry were distributed or not) relative to a line extending the head axis at the onset of the hunting behaviors was 46% and 54%, respectively. A generalized linear mixed model (GLMM) with a binomial error distribution and logit link function (individual ID was included as a random intercept to account for repeated measures within individuals) revealed no significant difference between them (estimate±s.e. 0.16±0.16, *z*=1.02, *P*=0.31; predicted probability of left-side 0.46 [95% CI: 0.38−0.54], right-side 0.54 [95% CI: 0.46−0.62]).

### Ontogenic changes in anatomically estimated visual field

Anatomical examination for the visual field was conducted for a total 50 specimens from 0 to 63 days of age (Fig. 4). At hatching, the lens plane of the hemispherical eye was inclined toward the arm, and this inclination was greater than that observed at later days of age.

The orientation of the lens plane on the hemispherical eye changed as the squid aged (Fig. 4C,D). Changes in eye orientation were expressed as logarithmic increases in the optical axis angle (Fig. 4Av,vii). A similar logarithmic inclination of the optical axis angle was observed as the squid grew (Fig. 4Avi,viii). In contrast, the uniocular visual field for both the left and right eyes increased linearly as the squid aged (Fig. 4Av,vii) and logarithmically as the squid grew (Fig. 4Avi,viii). Notably, the increase in the right eye was slightly greater than that in the left eye.

Although the binocular visual field was present anteriorly at 0 days old (11.45±6.22 deg; Fig. 4C), the anterior gap or overlap between the left and right uniocular visual fields was negligible for other ages. The visual field was calculated as the sum of the uniocular visual fields of the left and right eyes plus the gap or minus the overlap. Consequently, the maximum visual field widened markedly during the first week post-hatching and then remained constant (Fig. 4Aiii,C). A similar trend was observed in growth (Fig. 4Aiv). The visual field was estimated as 294.91±7.51 deg, 303.30 deg (mean±s.d., maximum) (5.96±0.26 mm ML) for 0 day old, 311.14±5.81 deg, 319.58 deg (7.57±0.44 mm ML) for 7 day old, 312.39±3.71 deg, 316.81 deg (8.68±0.51 mm ML) for 14 day old, 323.15±3.17 deg, 327.07 deg (11.25±1.09 mm ML) for 22 day old, 322.49±7.60 deg, 330.34 deg (14.76±0.84 mm ML) for 28 day old, 328.57±3.27 deg, 331.88 deg (16.21±1.10 mm ML) for 35 day-old, 322.65±5.80 deg, 331.87 deg (18.26±1.72 mm ML) for 42 day old, 323.52±2.91 deg, 326.38 deg (22.58±1.74 mm ML) for 49 day old, 322.51±1.42 deg, 324.77 deg (28.60±5.83 mm ML) for 57 day old and 323.06±7.31 deg, 329.28 deg (33.79±3.69 mm ML) for 63 day old squid (Fig. 4Aiii,iv).

## DISCUSSION

### Visual ontogeny of squid

In the present study, visual acuity estimated behaviorally showed a rapid increase immediately after hatching, whereas visual acuity estimated anatomically increased continuously during the post-hatching phase (Fig. 3Ai–iv). Differences in visual acuity estimated from behavioral and anatomical methods are common among a wide variety of animals possessing camera-type eyes (Dobson and Teller, 1978; Haug et al., 2010; Jones et al., 2007; Watanuki et al., 2000). The difference in this study may be attributed to the methodology used. Anatomically estimated visual acuity indicates the development of the theoretical resolving power of the eye. However, behaviorally estimated visual acuity is affected by multiple factors, including the development of the eye and optic lobes (regions of the brain used for visual processing) (Sakurai and Ikeda, 2022b), light sensitivity and the visual field. In particular, contrast sensitivity is one factor that strongly influences visual acuity as estimated from behavioral experiments. Because of their low contrast sensitivity, budgerigars showed similar values for target acuity and grating acuity, whereas humans can detect a single object far smaller than the grating acuity threshold (Chaib et al., 2019). While knowledge on cephalopod contrast sensitivity has been accumulated (Drerup et al., 2025), the relationship between contrast sensitivity and visual acuity remains unclear. A cuttlefish can hunt prey animals even under moonlight conditions (Brauckhoff et al., 2020), suggesting that squid are probably capable of hunting in extremely dark environments as well. Whether the squid, possessing relatively low contrast sensitivity like budgerigars, exhibit equivalent visual acuity in grating and target tests must be examined in the future through experiments with strictly controlled light conditions. Study on cephalopod visual acuity and visual field is still at an immature stage, therefore behavioral experiments tracking ontogenic processes presented here are particular challenges. In this study, we defined ‘squid visually detected prey’ as the onset of obvious hunting behavior, specifically when the squid rotated its head (not its eyes). However, it is impossible to completely rule out the possibility that the squid may shift its attention or preferences between detecting a specific prey and initiating pursuit, or even during the pursuit itself. This link failure might also relate to the differences in visual acuity estimated from behavioral and anatomical methods. Furthermore, it was considered that the low light intensity and non-uniform lighting in the experimental apparatus contributed to some of the variability observed in the behavioral experiment results.

As body size increased exponentially with age (Table 2), the ontogenic features of the lens size, which were an exponential increase with age (Fig. 3Av) and a linear increase with growth (Fig. 3Avi), show that the lens size probably increases in proportion to body size. In contrast, the retinal cell nuclei showed different ontogenic features, i.e. a linear decrease with age (Fig. 3Avii) and a logarithmic decrease with growth (Fig. 3Aviii) for maximum density and a logarithmic increase with both age (note the rapid increase until 14 days of age; Fig. 3Aix) and growth (Fig. 3Ax) for diameter. Therefore, the decrease in density of retinal cell nuclei was mainly induced by increases in cell nuclei diameter during the first 2 weeks post-hatching, and thereafter was probably mainly induced by the expanding retina, i.e. the increase in eye size, because the cell nuclei diameter reached a plateau (Fig. 3Avii–x, Bii,iv,vi). The increase in the rate of eye volume was reported to be smaller than the rate of growth during the 2 months post-hatching in oval squid (Sakurai and Ikeda, 2022a,b). This phenomenon is consistent with the results of the present study, which showed that the density of retinal cell nuclei markedly decreased linearly with body size after 2 weeks of age, and the slope of the regression line exhibited a slightly lower increasing rate of eye size than of growth rate (see the regression formula in Fig. 3Aviii). However, by comparing the slope of the regression lines between lens diameter and density of retinal cell nuclei against body size, the rate of increase in eye size was relatively larger than that in lens size (after 2 weeks of age; Fig. 3Avi,viii).

The highest-density region of the retinal cell nuclei in the posterior and slightly dorsal or middle areas of the retina (Fig. 3C) showed that the visual axis of younger squid was directed downward and forward, similar to that of older squid (Makino and Miyazaki, 2010). In the present study, the shift in the range for density of retinal cell nuclei (×10^2^ cells mm^−2^) in the iso-density map from 260 to 300 (41 to 48 days old) to 200 to 260 (55 to 62 days old) corresponded to the decrease in the maximum density of retinal cell nuclei (Figs 3Avii,viii and 3C). Because this downward trend is similar to the density of retinal cell nuclei at 140–300 (×10^2^ cells mm^−2^) for older squid (Makino and Miyazaki, 2010), it would be safe to say that the decrease in the density of retinal cell nuclei, especially in the peripheral area of the retina (i.e. closer to the lens plane of the eye), along with growth must be a predominant characteristic.

The finding that visual acuity increased despite the decreasing density of retinal cell nuclei shows that visual acuity is affected strongly by lens size (Fig. 3Aiii–viii, Bii,iv,vi). This relationship also showed a similar exponential increase with age and a linear increase with body size for both visual acuity and lens size (Fig. 3Aiii–vi). These facts are consistent with the property of the formula we employed in the anatomical examination that the visual acuity decreases if the density of retinal cell nuclei decreases and is a function of body and lens sizes. However, the shallow slope of the regression lines for lens diameter and visual acuity indicated an increase in rate lower than that for body size, i.e. the growth rate (Fig. 3Avi,iv). This is consistent with previous findings that the volume of the eyes increases more slowly than body size does (Sakurai and Ikeda, 2022b). By clarifying the relationship between visual acuity and the optic properties of lenses which directly determine their focusing power (Sweeney et al., 2007), future studies may yield a deeper understanding of the ontogenic process of visual acuity.

Whereas the development of the resolving power of the eye also increases behaviorally with estimated visual acuity, the development of optic lobes and light sensitivity accelerates that increase immediately after hatching. The volume of the optic lobes increases in proportion to body size but more slowly (Sakurai and Ikeda, 2022b). The rapid development of light sensitivity during the early phase post-hatching was shown as a liner increase with aging (Fig. 3Axi) and a logarithmic increase with growth (Fig. 3Axii) of the maximum height of the rhabdomeric layer in this study. The rapid increase of the diameter of retinal cell nuclei may also relate to the rapid development of light sensitivity if there is a correlation between the diameter of the distal segment of retinal cell and the size of retinal cell nuclei (Fig. 3Aix,x).

In contrast to the difference in the ontogenic features of visual acuity between behavioral and anatomical examination, the visual field exhibited a remarkable increase immediately after hatching in both examinations. In the behavioral examination, the expansion of the visual field occurred slightly earlier (up to 1 week old) than increases in visual acuity (up to 2 weeks old). This may indicate that the visual field accelerates the increase of behaviorally estimated visual acuity immediately after hatching. The finding that the rate of increase for the visual field behaviorally was larger than that anatomically (Fig. 4Ai–iv,B,C) is related to the actions of the extraocular eye muscles. In cephalopods, muscles connect to the eye, by which each eye can be rotated independently, contributing to binocular vision (Budelmann and Young, 1984, 1993; Packard, 1972). In our anatomical examination, all data were collected from anesthetized specimens. Because ethanol (for anesthesia) blocks nerve impulse transmission and annulus reflex arcs, the specimens were prevented from exercising (Brady and Carbone, 1973; García-Franco, 1992; Ikeda et al., 2009). Hence, the extraocular eye muscles were also prevented from acting and thus malfunctioned during eye rotation. However, for behavioral examination, the extraocular eye muscles must work for eye movements, in which the squid has an enlarged visual field. Because the 0 day old individuals showed a narrower visual field in the behavioral examination than in the anatomical examination, the extraocular eye muscles probably acted soon after hatching (Fig. 4B,C). This is comparable to other muscles, such as the tentacles, which are immature at hatching and then develop as the squid grows (Kier, 1996). The oval squid employs binocular vision for hunting soon after hatching, which is generally used in the older phases in squid and cuttlefish (Budelmann and Young, 1984, 1993; Feord et al., 2020).

However, the visual field showed a large difference between behavioral and anatomical examination for hatchlings and 63 day old squid (Fig. 4Ai–iv,B,C). This discrepancy is partially explained by the motivation to hunt. For hatchlings, because the inner yolk acts as a backup ration until they develop hunting skills (Chen et al., 1996), hatchlings might have a lower motivation for hunting (i.e. fewer attacks) than older squid (Fig. 4B and Table 3). Oval squid begin to form schools after 1 month of age (Sugimoto and Ikeda, 2012), from which they probably gain social conditioning. The 63 day old squid showed extremely few attacks (Fig. 4B and Table 3), which might be because the subject squid were nervous owing to the solitary situation in the experimental apparatus. The decrease in the number of attacks by squid after 42 days of age may also be due to stress arising from the same condition. Variation in results from behavioral experiments stems from numerous factors, including fluctuations in individual internal states (such as the difficulty of determining which prey is detected by which eye) and even slight differences in experimental apparatus, such as precise lighting conditions. It is impossible to completely avoid these factors.

The ontogenic change of the visual field was anatomically detected as the change of the eye orientation (Fig. 4C,D). The eye orientation, i.e. the orientation of the lens plane on the hemispherical eye, which was expressed as the optical axis angle, showed a logarithmic increase with age (Fig. 4Av,vii) and with growth (Fig. 4Avi,viii) for both the left and right eyes. This result indicated that the lens plane on the hemispherical eye inclined toward the arm at hatching and was nearly parallel to the head axis at 7 days of age (Fig. 4D). In contrast, the uniocular visual field for both the left and right eyes showed a linear increase with age (Fig. 4Av,vii) and a logarithmic increase with growth (Fig. 4Avi,viii). Enlargement of the uniocular visual field, which is the angle between the anterior pupillary axis (from the posterior retinal edge to the anterior lens edge) and the posterior axis (from the anterior retinal edge to the posterior lens edge) (Fig. 2), is induced by enlargement of the lens diameter and expansion of the retina, i.e. an increase in eye size. Because the increase in eye size (estimated by retinal cell nuclei density) was relatively larger than the increase in lens size against body size (Fig. 3Avi,viii), eye size must be more influential on the uniocular visual field than lens size.

The maximum visual field showed a logarithmic increase with age (Fig. 4Aiii,C) and with growth (Fig. 4Aiv). Because rapid enlargement of the visual field was detected during the same period as a rapid increase in the optical axis angle for both eyes, eye orientation must strongly contribute to the enlargement of the visual field until 3 weeks old, and then cease (Fig. 4Aiii–viii,C,D). However, an increase in eye size is reflected by a continuous increase in the uniocular visual field. This phenomenon continued throughout the experimental period (Fig. 4Av–viii); therefore, except for the first several weeks post-hatching, the visual field might keep increasing slightly as the eye grows.

Although the binocular visual field was clearly identified only in the anterior region at hatching based on anatomical examination (Fig. 4C), squid besides hatchlings would also generate this visual field using the extraocular eye muscles. In general, squid use binocular vision for hunting (Budelmann and Young, 1984, 1993; Feord et al., 2020). The higher density of retinal cells in the posterior region of the retina suggests that squid can see more clearly in the anterior than in the posterior region (Fig. 3C). Although the posterior binocular visual field was not observed, the possibility of its usage remains because of extraocular eye muscles and ontogenic changes in lens and eyeball sizes that were estimated by the relationship between the density and diameter of retinal cell nuclei (Fig. 3Aiii–x). Because 14, 22, 28, 49 and 57 day old squid showed a higher maximum visual field in the behavioral examination than in the anatomical examination, the extraocular eye muscles would also act in the posterior binocular visual field (Fig. 4B,C). A slight but continuous enlargement of the uniocular visual field probably also helps to enlarge the posterior binocular visual field (Fig. 4Av–viii).

### Comparison of visual ontogeny of camera-type eyes

This study uncovered the ontogenic process of vision derived from visual acuity and the visual field, and examined it behaviorally as well as anatomically, for the first time in cephalopods. The results provide an overview of ontogenic features related to vision among cephalopods (Table 4). Estimating the ontogenic process of the visual field based on images of the inclination of the eyes toward their head axis (Table 4) is a novel method in the present study. The results indicate that the rapid increase in both visual acuity and visual field immediately after hatching is a common feature of cephalopods.

**Table 4.** Comparison for ontogeny of visual acuity in cephalopods

Squid (*Sepioteuthis lessoniana*)			Cuttlefish (*Sepia officinalis*)			Octopus (*Octopus vulgaris*)		
Mantle length (mm)^a^	Visual acuity (cpd)^a^	Visual field (deg)^b^	Mantle length (mm)^1^	Visual acuity (cpd)^1,d^	Visual field^c^	Body mass (g)^2^	Visual acuity (cpd)^2,d^	Visual field^c^
Hatchling (6.2)	0.63	295	Hatchling^5^ (10)	∼0.20	May be wide^7^	Hatchling (1.4 mg, 2.0 mm)^9^	−	May be narrow^11^
7 days old (5.5)	0.65	311	−	−	−	15 days old (4.2 mg, 2.9 mm)^9^	−	−
14 days old (9.8)	0.90	312	−	−	−	30 days old (17.4 mg, 4.5 mm)^9^	0.12^9^	−
22 days old (11.3)	1.11	323	−	−	−	42 days old (40.3 mg, 6.4 mm)^9^	−	−
28 days old (13.2)	1.18	322	20	∼0.40	−	60 days old (173.2 mg, 8.6 mm)^9^	−	May be wide^12^
35 days old (13.8)	0.78	329	40	∼0.50	−	−	−	−
42 days old (19.4)	1.20	323	80	0.88	−	0.5	< 0.61	−
49 days old (22.0)	1.26	324	−	−	−	2.7	0.61	−
57 days old (25.8)	1.26	323	−	−	−	17.0	1.11	−
63 days old (40.2)	2.01	323	−	−	−	−	−	−
May be adult^3^ (180.0^4^)	7.63^4,d^	−	Adult (unknown size)^6^	6.66^6^	360 deg <^8^	May be adult (500.0)^10^	1.76^10^	−

^a^Behavioral experiment in the present study. ^b^Anatomical experiment in the present study. ^c^Estimation of the inclination of the eyes toward the head axis in the images. ^d^Visual acuity estimates of these studies were recalculated using our method with the available data. Data for 10−40 mm *S. officinalis* were estimated from the graph in Groeger et al. (2005). ^1^Groeger et al. (2005). ^2^Packard (1969). ^3^Nabhitabhata and Ikeda (2014). ^4^Makino and Miyazaki (2010). ^5^Boletzky (1983). ^6^Schaeffel et al. (1999). ^7^Navet et al. (2017) and Bonadé et al. (2020). ^8^Messenger (1968). ^9^Villanueva et al. (1996). ^10^Sutherland (1963). ^11^Deryckere et al. (2020). ^12^Roura et al. (2023).

The rapid increase in the visual field occurred slightly earlier than that in visual acuity in the oval squid (Figs 3Ai–iv and 4Ai–iv). This observation also applies to cuttlefish and octopus (Table 4). During the post-hatching phase, squid and octopus are planktonic, whereas cuttlefish are benthic. As juveniles are unable to move adequately immediately after hatching, it is advantageous for them to see across wider but closer areas to detect prey. This is particularly true for planktonic juveniles exposed to water columns. Oval squid employ hunting behavior with their entire arms from a close distance during the early life phase, whereas they employ tentacles to hunt from further distances at older ages (Sugimoto and Ikeda, 2013). The same is true for cuttlefish, as they employ tentacular hunting just after hatching, but the distance to initiate hunting is shorter than that at older ages (Sugimoto and Ikeda, 2013). Generally, cephalopods require higher amounts of nutrients during their early life phase because starvation is the main cause of death during this phase (Ikeda et al., 2005). Because cephalopods lack parental care, they must obtain their diet themselves in the post-hatching phase. Therefore, detecting prey is a survival priority for cephalopod hatchlings. In addition, newly hatched juveniles are usually small and incapable of moving quickly over long distances (Villanueva et al., 2016). In such situations, an adaptive strategy would be to have a wide visual field, even with low visual acuity (i.e. myopia). This vision feature during the early life phase seems to be a shared feature among cephalopods (Table 4) despite species-specific differences for other aspects of vision [e.g. existence or lack of densely occurring retinal cells (Talbot and Marshall, 2010; Watanuki et al., 2000) and number of extraocular eye muscles (Budelmann and Young, 1984, 1993)].

The eyes in cephalopod hatchlings are smaller than the optic lobes, a region of the brain used in visual processing, thus pushing each eye toward the arm (Fig. 4D; see images in Navet et al., 2017; Roura et al., 2023; Vijai et al., 2015). This is the case in the oval squid; however, the relationship between these two organs is reversed late in the post-hatching phase (Fig. 4D; Sakurai and Ikeda, 2022b). The timing of this switch between the eye and optic lobe sizes (Sakurai and Ikeda, 2022b) was consistent with the rapid widening of the visual field (Fig. 4Ai–iv). This finding tentatively indicates that a wider visual field is accomplished by inclination of the lens plane (Fig. 4D) and rotation of the eyeballs by the extraocular eye muscles. However, the development of visual acuity takes time because the speed of lens enlargement is restricted by the slower growth of the body, including of the head region (Fig. 3Ai–xii).

The visual acuity of humans reaches the adult level at 5 years of age (Chandna, 1991) or takes a few more years (Leat et al., 2009). In other mammals, similar attainment occurs within 5–6 weeks (monkey, *Macaca nemestrina*; Mitchell et al., 1976) or 3–4 months (cat; Teller et al., 1978) after birth. In chickens, maximum visual acuity is achieved within 2 days of hatching (Over and Moore, 1981) or after a few days more (Schmid and Wildsoet, 1998). In fishes, visual acuity shows a logarithmic increase after hatching [*Alosa pseudoharengus*, *Perca flavescens*, *Coregonus hoyi* (Miller et al., 1993); *Apogon doederleini*, *Stethojulis strigiventer*, *Upeneus tragula*, *Pomacentrus moluccensis* (Shand, 1997); *Perca flavescens*, *Rutilus rutilus* (Wanzenböck et al., 1996)] whereas the eye keeps growing in terms of retinal genesis and lenticular growth [*Carassius auratus* (Johns and Easter, 1977)].

In contrast to visual acuity, knowledge of the ontogeny of the visual field is limited to vertebrates. The visual field of humans reaches the adult extent at 6 months or 1 year of age, depending on the test procedure (Lewis and Maurer, 1992; Sireteanu, 1996). Regarding other mammals, it is reached after 8–10 weeks of age in cats and may be similar in monkeys (Sireteanu, 1996). In fishes, the range of the visual field changes in response to feeding behavior during metamorphosis (Collin and Shand, 2003).

In short, the development of visual acuity and the visual field is concentrated during the early life phase in mammals, birds and fishes. Furthermore, the visual field develops faster than visual acuity in mammals and birds. This trend is also common among cephalopods, meaning that although cephalopod retinas are non-converted and possess a structure entirely distinct from the converted retinas of vertebrates, they share this characteristic feature of camera-type eyes.

However, the practical duration necessary for the development of visual acuity and the visual field differs between these vertebrates and cephalopods. Because parental care (mammals and birds) compensates for the vulnerability of juveniles after birth, it can provide an extended time for juveniles to develop their vision. In fishes, metamorphosis in response to habitat shifts provides an opportunity to develop vision, which improves their foraging and predator avoidance capabilities (Beaudet and Hawryshyn, 1999). However, because cephalopods lack both parental care and metamorphosis, they need to facilitate vision for offence and defense within a short duration after birth. This may cause a difference in the time to vision development between vertebrates (slower) and cephalopods (faster) during the early life phase.

## References

[JEB250437C1] Beaudet, L. and Hawryshyn, C. W. (1999). Ecological aspects of vertebrate visual ontogeny. In *Adaptive Mechanisms in the Ecology of Vision* (ed. S. N. Archer, M. B. A. Djamgoz, E. R. Loew, J. C. Partridge and S. Vallerga), pp. 413-437. New York: Springer. 10.1007/978-94-017-0619-3

[JEB250437C2] Boletzky, S. V. (1974). The ‘larvae’ of Cephalopoda: a review. *Thalass. Jugoslavica* 10, 45-76.

[JEB250437C3] Boletzky, S. V. (1983). Sepia officinalis. In *Cephalopod Life Cycles* (ed. P. R. Boyle), pp. 31-52. Cambridge, MA: Academic Press.

[JEB250437C4] Bonadè, M., Ogura, A., Corre, E., Bassaglia, Y. and Bonnaud-Ponticelli, L. (2020). Diversity of light sensing molecules and their expression during the embryogenesis of the cuttlefish (*Sepia officinalis*). *Front. Physiol.* 11, 521989. 10.3389/fphys.2020.521989 33117186 PMC7553075

[JEB250437C5] Bozzano, A., Pankhurst, P. M., Moltschaniwskyj, N. A. and Villanueva, R. (2009). Eye development in southern calamary, *Sepioteuthis australis*, embryos and hatchlings. *Mar. Biol.* 156, 1359-1373. 10.1007/s00227-009-1177-2

[JEB250437C6] Brady, R. O. and Carbone, E. (1973). Comparison of the effects of Δ9-tetrahydrocannabinol, 11-hydroxy-Δ9-tetrahydrocannabinol, and ethanol on the electrophysiological activity of the giant axon of the squid. *Neuropharmacology* 12, 601-605. 10.1016/0028-3908(73)90010-5 4725527

[JEB250437C87] Brauckhoff, M., Wahlberg, M., Haga, J. Å. R., Karlsen, H. E. and Wilson, M. (2020). Embracing their prey at that dark hour: common cuttlefish (*Sepia officinalis*) can hunt in nighttime light conditions. *Front. Physiol.* 11, 525. 10.3389/fphys.2020.00525 32587521 PMC7298144

[JEB250437C7] Brown, P. K. and Brown, P. S. (1958). Visual pigments of the octopus and cuttlefish. *Nature* 182, 1288-1290. 10.1038/1821288a0 13600294

[JEB250437C8] Budelmann, B. U. and Young, J. Z. (1984). The statocyst-oculomotor system of *Octopus vulgaris*: extraocular eye muscles, eye muscle nerves, statocyst nerves and the oculomotor centre in the central nervous system. *Philos. Trans. R. Soc. Lond. B. Biol. Sci.* 306, 159-189. 10.1098/rstb.1984.0084 8099747

[JEB250437C9] Budelmann, B. U. and Young, J. Z. (1993). The oculomotor system of decapod cephalopods: eye muscles, eye muscle nerves, and the oculomotor neurons in the central nervous system. *Philos. Trans. R. Soc. Lond. B. Biol. Sci.* 340, 93-125. 10.1098/rstb.1993.0051 8099747

[JEB250437C10] Caves, E. M., Brandley, N. C. and Johnsen, S. (2018). Visual acuity and the evolution of signals. *Trends Ecol. Evol.* 33, 358-372. 10.1016/j.tree.2018.03.001 29609907

[JEB250437C11] Chaib, S., Ljungholm, M., Lind, O. and Kelber, A. (2019). Single target acuity is not higher than grating acuity in a bird, the budgerigar. *Vision Res.* 160, 37-42. 10.1016/j.visres.2019.04.005 31075286

[JEB250437C12] Chandna, A. (1991). Natural history of the development of visual acuity in infants. *Eye* 5, 20-26. 10.1038/eye.1991.4 2060665

[JEB250437C13] Chen, D. S., Van Dykhuizen, G., Hodge, J. and Gilly, W. F. (1996). Ontogeny of copepod predation in juvenile squid (*Loligo opalescens*). *Biol. Bull.* 190, 69-81. 10.2307/1542676 8852631

[JEB250437C88] Chung, W. S. and Marshall, N. J. (2017). Complex visual adaptations in squid for specific tasks in different environments. *Front. Physiol.* 8, 105. 10.3389/fphys.2017.00105 28286484 PMC5323406

[JEB250437C14] Collin, S. P. and Pettigrew, J. D. (1989). Quantitative comparison of the limits on visual spatial resolution set by the ganglion cell layer in twelve species of reef teleosts. *Brain. Behav. Evol.* 34, 184-192. 10.1159/000116504 2590834

[JEB250437C15] Collin, S. P. and Shand, J. (2003). Retinal sampling and the visual field in fishes. In *Sensory Processing in Aquatic Environments* (ed. N. J. Collin and S. P. Marshall), pp. 139-169. New York: Springer.

[JEB250437C16] Croft, D. P., James, R., Thomas, P. O. R., Hathaway, C., Mawdsley, D., Laland, K. N. and Krause, J. (2006). Social structure and co-operative interactions in a wild population of guppies (*Poecilia reticulata*). *Behav. Ecol. Sociobiol.* 59, 644-650. 10.1007/s00265-005-0091-y

[JEB250437C17] Deryckere, A., Styfhals, R., Vidal, E. A. G., Almansa, E. and Seuntjens, E. (2020). A practical staging atlas to study embryonic development of *Octopus vulgaris* under controlled laboratory conditions. *BMC Dev. Biol.* 20, 7. 10.1186/s12861-020-00212-6 32299349 PMC7164171

[JEB250437C18] Dobson, V. and Teller, D. Y. (1978). Visual acuity in human infants: a review and comparison of behavioral and electrophysiological studies. *Vision Res.* 18, 1469-1483. 10.1016/0042-6989(78)90001-9 364823

[JEB250437C19] Drerup, C., How, M. J. and Herbert-Read, J. E. (2025). Visual contrast from background features and dynamic illumination contributes to three-dimensional camouflage in cuttlefish. *J. Exp. Biol.* 228, jeb249713. 10.1242/jeb.249713 40814832 PMC12401539

[JEB250437C20] Feord, R. C., Sumner, M. E., Pusdekar, S., Kalra, L., Gonzalez-Bellido, P. T. and Wardill, T. J. (2020). Cuttlefish use stereopsis to strike at prey. *Sci. Adv.* 6, eaay6036. 10.1126/sciadv.aay6036 31934631 PMC6949036

[JEB250437C21] Fernald, R. D. (2006). Casting a genetic light on the evolution of eyes. *Science* 313, 1914-1918. 10.1126/science.1127889 17008522

[JEB250437C22] Froesch, D. (1973). On the fine structure of the octopus iris. *Z. Zellforsch. Mikrosk. Anat.* 145, 119-129. 10.1007/BF00307193 4360461

[JEB250437C23] García-Franco, M. (1992). Anaesthetics for the squid *Sepioteuthis sepioidea* (Mollusca: Cephalopoda). *Comp. Biochem. Physiol.* 103, 121-123. 10.1016/0742-8413(92)90239-4

[JEB250437C24] Groeger, G., Cotton, P. A. and Williamson, R. (2005). Ontogenetic changes in the visual acuity of *Sepia officinalis* measured using the optomotor response. *Can. J. Zool.* 83, 274-279. 10.1139/z05-011

[JEB250437C25] Hajar, M. A. I., Inada, H., Hasobe, M. and Arimoto, T. (2008). Visual acuity of Pacific saury *Cololabis saira* for understanding capture process. *Fish. Sci.* 74, 461-468. 10.1111/j.1444-2906.2008.01547.x

[JEB250437C26] Hanlon, R. T. and Messenger, J. B. (2018). *Cephalopod Behaviour*. Cambridge, UK: Cambridge University Press. 10.1017/9780511843600

[JEB250437C27] Hao, Z. L., Zhang, X. M., Kudo, H. and Kaeriyama, M. (2010). Development of the retina in the cuttlefish *Sepia esculenta*. *J. Shellfish Res.* 29, 463-470. 10.2983/035.029.0224

[JEB250437C28] Haug, M. F., Biehlmaier, O., Mueller, K. P. and Neuhauss, S. C. F. (2010). Visual acuity in larval zebrafish: behavior and histology. *Front. Zool.* 7, 8. 10.1186/1742-9994-7-8 20193078 PMC2848032

[JEB250437C29] Ikeda, Y., Sakurazawa, I., Ito, K., Sakurai, Y. and Matsumoto, G. (2005). Rearing of squid hatachlings, *Heterololigo bleekeri* (Keferstein 1866) up to 2 months of a closed system. *Aquac. Res.* 36, 409-412. 10.1111/j.1365-2109.2005.01217.x

[JEB250437C30] Ikeda, Y., Sugimoto, C., Yonamine, H. and Oshima, Y. (2009). Method of ethanol anaesthesia and individual marking for oval squid (*Sepioteuthis lessoniana* Férussac, 1831 in Lesson 1830-1831). *Aquac. Res.* 41, 157-160. 10.1111/j.1365-2109.2009.02305.x

[JEB250437C31] Imai, H. and Aoki, M. (2012). Genetic diversity and genetic heterogeneity of bigfin reef squid “*Sepioteuthis lessoniana*” species complex in northwestern Pacific ocean. In *Analysis of Genetic Variation in Animals* (ed. M. Caliskan). InTech. 10.5772/35024

[JEB250437C32] Izuka, T., Segawa, S., Okutani, T. and Numachi, K. (1994). Evidence on the existence of three species in the oval squid *Sepioteuthis lessoniana* Complex in Ishigaki Island, Okinawa, Southwestern Japan, by Isozyme Analyses. *Venus: Jap. J. Malacol.* 53, 217-228.

[JEB250437C33] Izuka, T., Segawa, S. and Okutani, T. (1996). Identification of three species in oval squid, *Sepioteuthis lessoniana* complex by chromatophore arrangements on the funnel. *Venus: Jap. J. Malacol.* 55, 139-142.

[JEB250437C34] Jereb, P. and Roper, C. F. E. (2006). Cephalopods of the Indian ocean. A review. Part I. Inshore squids (Loliginidae) collected during the Ineternational Indian Ocean expedition*. Proc. Biol. Soc. Washingt.* 119, 91-136. 10.2988/0006-324X(2006)119[91:COTIOA]2.0.CO;2

[JEB250437C35] Johns, P. R. and Easter, S. S. (1977). Growth of the adult goldfish eye-II: increase in retinal cell number. *Vision Res.* 17, 469-477. 10.1016/0042-6989(77)90041-4 878338

[JEB250437C36] Jones, M. P., Pierce, K. E. and Ward, D. (2007). Avian vision: a review of form and function with special consideration to birds of prey. *J. Exot. Pet Med.* 16, 69-87. 10.1053/j.jepm.2007.03.012

[JEB250437C37] Kier, W. M. (1996). Muscle development in squid: ultrastructural differentiation of a specialized muscle fiber type. *J. Morphol.* 229, 271-288. 10.1002/(SICI)1097-4687(199609)229:3<271::AID-JMOR3>3.0.CO;2-1 29852595

[JEB250437C38] Land, M. F. (2012). The evolution of lenses. *Ophthalmic Physiol. Opt.* 32, 449-460. 10.1111/j.1475-1313.2012.00941.x 23057564

[JEB250437C39] Leat, S. J., Yadav, N. K. and Irving, E. L. (2009). Development of visual acuity and contrast sensitivity in children. *J. Optom.* 2, 19-26. 10.3921/joptom.2009.19

[JEB250437C40] Lewis, T. L. and Maurer, D. (1992). The development of the temporal and nasal visual fields during infancy. *Vision Res.* 32, 903-911. 10.1016/0042-6989(92)90033-F 1604859

[JEB250437C41] Makino, A. and Miyazaki, T. (2010). Topographical distribution of visual cell nuclei in the retina in relation to the habitat of five species of decapodiformes (Cephalopoda). *J. Molluscan Stud.* 76, 180-185. 10.1093/mollus/eyp055

[JEB250437C42] Matsui, S., Seidou, M., Horiuchi, S., Uchiyama, I. and Kito, Y. (1988). Adaptation of a deep-sea cephalopod to the photic environment. *J. Gen. Physiol.* 92, 55-66. 10.1085/jgp.92.1.55 3171534 PMC2228886

[JEB250437C43] Messenger, J. B. (1968). Visual attack of cuttlefish *Sepia officinalis*. *Anim. Behav.* 16, 342-357 10.1016/0003-3472(68)90020-1 5691850

[JEB250437C44] Miller, T. J., Crowder, L. B. and Rice, J. A. (1993). Ontogenetic changes in behavioural and histological measures of visual acuity in three species of fish. *Environ. Biol. Fishes* 37, 1-8. 10.1007/BF00000707

[JEB250437C45] Mitchell, D. E., Giffin, F., Wilkinson, F., Anderson, P. and Smith, M. L. (1976). Visual resolution in young kittens. *Vision Res.* 16, 363-366. 10.1016/0042-6989(76)90197-8 941412

[JEB250437C46] Nabhitabhata, J. and Ikeda, Y. (2014). Sepioteuthis lessoniana. In *Cephalopod Culture* (ed. J. Iglesias, L. Fuentes and R. Villanueva), pp. 315-347. New York: Springer. 10.1007/978-94-017-8648-5

[JEB250437C47] Napoli, F. R., Daly, C. M., Neal, S., McCulloch, K. J., Zaloga, A. R., Liu, A. and Koenig, K. M. (2022). Cephalopod retinal development shows vertebrate-like mechanisms of neurogenesis. *Curr. Biol.* 32, 5045-5056.e3. 10.1016/j.cub.2022.10.027 36356573 PMC9729453

[JEB250437C48] Navet, S., Buresi, A., Baratte, S., Andouche, A., Bonnaud-Ponticelli, L. and Bassaglia, Y. (2017). The Pax gene family: highlights from cephalopods. *PLoS ONE* 12, e0172719. 10.1371/journal.pone.0172719 28253300 PMC5333810

[JEB250437C49] Nilsson, D. E., Warrant, E. J., Johnsen, S., Hanlon, R. and Shashar, N. (2012). A unique advantage for giant eyes in giant squid. *Curr. Biol.* 22, 683-688. 10.1016/j.cub.2012.02.031 22425154

[JEB250437C50] Over, R. and Moore, D. (1981). Spatial acuity of the chicken. *Brain Res.* 211, 424-426. 10.1016/0006-8993(81)90967-7 7237130

[JEB250437C51] Packard, A. (1969). Visual acuity and eye growth in *Octopus vulgaris* (Lamarck). *Monit. Zool. Ital.* 3, 19-32.

[JEB250437C52] Packard, A. (1972). Cephalopods and fish: the limits of convergence. *Biol. Rev.* 47, 241-307. 10.1111/j.1469-185x.1972.tb00975.x

[JEB250437C53] Partridge, J. C. (2012). Sensory ecology: giant eyes for giant predators? *Curr. Biol.* 22, R268-R270. 10.1016/j.cub.2012.03.021 22537628

[JEB250437C54] Pichaud, F. and Casares, F. (2022). Shaping an optical dome: the size and shape of the insect compound eye. *Semin. Cell Dev. Biol.* 130, 37-44. 10.1016/j.semcdb.2021.11.002 34810110

[JEB250437C55] Roura, A., Castro-Bugallo, A. and Martínez-Pérez, M. (2023). The settlement stage in the common octopus *Octopus vulgaris* Cuvier, 1797: a complex transition between planktonic and benthic lifestyles. *Mar. Biol.* 170, 1-12. 10.1007/s00227-023-04188-2

[JEB250437C56] Saibil, H. R. (1990). Structure and function of the squid eye. In *Squid as Experimental Animals* (ed. D. L. Gilbert, W. J. Adelman and J. M. Arnold), pp. 371-397. New York: Springer. 10.1007/978-1-4899-2489-6

[JEB250437C57] Sakurai, Y. and Ikeda, Y. (2022a). Visual and brain lateralization during the posthatching phase in squid under solitary and group conditions. *Anim. Behav.* 183, 13-28. 10.1016/j.anbehav.2021.10.015

[JEB250437C58] Sakurai, Y. and Ikeda, Y. (2022b). Allometry for eyes and optic lobes in oval squid (*Sepioteuthis lessoniana*) with special reference to their ontogenetic asymmetry. *Symmetry (Basel)*. 14, 644. 10.3390/sym14040644

[JEB250437C59] Schaeffel, F., Murphy, C. J. and Howland, H. C. (1999). Accommodation in the cuttlefish (*Sepia officinalis*). *J. Exp. Biol.* 202, 3127-3134. 10.1242/jeb.202.22.3127 10539961

[JEB250437C60] Schmid, K. L. and Wildsoet, C. F. (1998). Assessment of visual acuity and contrast sensitivity in the chick using an optokinetic nystagmus paradigm. *Vision Res.* 38, 2629-2634. 10.1016/S0042-6989(97)00446-X 12116708

[JEB250437C61] Segawa, S., Hirayama, S. and Okutani, T. (1993a). Is *Sepioteuthis lessoniana* in Okinawa a single species? In *Recent Advances in Cephalopod Fisheries Biology* (ed. T. Okutani, R. K. O'Dor and T. Kubodera), pp. 513-521. Kanagawa, Japan: Tokai University Press.

[JEB250437C62] Segawa, S., Izuka, T., Tamashiro, T. and Okutani, T., Hamanaka, T., Michinomae, M., Yoshihara, K. and Kito, Y. (1993b). A note on mating and egg deposition by *Sepioteuthis lessoniana* in Ishigaki Island, Okinawa, Southwestern Japan. *Venus Jap. J. Malacol.* 52, 101-108. 10.18941/venusjjm.52.1_101

[JEB250437C63] Seidou, M., Sugahara, M., Uchiyama, H., Hiraki, K., Hamanaka, T., Michinomae, M., Yoshihara, K. and Kito, Y. (1990). On the three visual pigments in the retina of the firefly squid, *Watasenia scintillans*. *J. Comp. Physiol. A* 166, 769-773. 10.1007/BF00187321

[JEB250437C64] Shand, J. (1997). Ontogenetic changes in retinal structure and visual acuity: a comparative study of coral-reef teleosts with differing post-settlement lifestyles. *Environ. Biol. Fishes* 49, 307-322. 10.1023/A:1007353003066

[JEB250437C65] Sireteanu, R. (1996). Development of the visual field: results from human and animal studies. In *Infant Vision* (ed. F. Vital-Durand, J. Atkinson and O. J. Braddick), pp. 17-31. Oxford, UK: Oxford University Press.

[JEB250437C66] Smith, J. A., Andrews, P. L. R., Hawkins, P., Louhimies, S., Ponte, G. and Dickel, L. (2013). Cephalopod research and EU Directive 2010/63/EU: requirements, impacts and ethical review. *J. Exp. Mar. Bio. Ecol.* 447, 31-45. 10.1016/j.jembe.2013.02.009

[JEB250437C67] Sugimoto, C. and Ikeda, Y. (2012). Ontogeny of schooling behavior in the oval squid *Sepioteuthis lessoniana*. *Fish. Sci.* 78, 287-294. 10.1007/s12562-011-0464-2

[JEB250437C68] Sugimoto, C. and Ikeda, Y. (2013). Comparison of the ontogeny of hunting behavior in pharaoh cuttlefish (*Sepia pharaonis*) and oval squid (*Sepioteuthis lessoniana*). *Biol. Bull.* 225, 50-59. 10.1086/BBLv225n1p50 24088796

[JEB250437C69] Sutherland, N. S. (1963). Visual acuity and discrimination of stripe widths in *Octopus vulgaris* Lamarck. *Pubbl. Stn. Zool. Napoli* 33, 92-109.

[JEB250437C70] Sweeney, A. M., Haddock, S. H. D. and Johnsen, S. (2007). Comparative visual acuity of coleoid cephalopods. *Integr. Comp. Biol.* 47, 808-814. 10.1093/icb/icm092 21669760

[JEB250437C71] Talbot, C. M. and Marshall, J. (2010). Polarization sensitivity and retinal topography of the striped pyjama squid (*Sepioloidea lineolata* -Quoy/Gaimard 1832). *J. Exp. Biol.* 213, 3371-3377. 10.1242/jeb.048165 20833931

[JEB250437C72] Talbot, C. M. and Marshall, J. N. (2011). The retinal topography of three species of coleoid cephalopod: significance for perception of polarized light. *Philos. Trans. R. Soc. B Biol. Sci.* 366, 724-733. 10.1098/rstb.2010.0254 PMC3049017 21282176

[JEB250437C73] Tamura, T. (1957). A study of visual perception in fish, especially on resolving power and accommodation. *Bull. Jap. Soc. Sci. Fish.* 22, 536-557. 10.2331/suisan.22.536

[JEB250437C74] Teller, D. Y., Regal, D. M., Videen, T. O. and Pulos, E. (1978). Development of visual acuity in infant monkeys (*Macaca nemestrina*) during the early postnatal weeks. *Vision Res.* 18, 561-566. 10.1016/0042-6989(78)90203-1 96592

[JEB250437C75] Torisawa, S., Takagi, T., Ishibashi, Y., Sawada, Y. and Yamane, T. (2007). Changes in the retinal cone density distribution and the retinal resolution during growth of juvenile Pacific bluefin tuna *Thunnus orientalis*. *Fish. Sci.* 73, 1202-1204. 10.1111/j.1444-2906.2007.01454.x

[JEB250437C76] Vijai, D., Sakai, M. and Sakurai, Y. (2015). Embryonic and paralarval development following artificial fertilization in the neon flying squid *Ommastrephes bartramii*. *Zoomorphology* 134, 417-430. 10.1007/s00435-015-0267-6

[JEB250437C77] Villanueva, R., Nozais, C. and Boletzky, S. V. (1996). Swimming behaviour and food searching in planktonic *Octopus vulgaris* Cuvier from hatching to settlement. *J. Exp. Mar. Bio. Ecol.* 208, 169-184. 10.1016/S0022-0981(96)02670-6

[JEB250437C78] Villanueva, R., Vidal, E. A. G., Fernández-Álvarez, F. Á. and Nabhitabhata, J. (2016). Early mode of life and hatchling size in cephalopod molluscs: influence on the species distributional ranges. *PLoS ONE* 11, e0165334. 10.1371/journal.pone.0165334 27829039 PMC5102429

[JEB250437C79] Wanzenböck, J. and Schiemer, F. (1989). Prey detection in cyprinids during early development. *Can. J. Fish. Aquat. Sci.* 46, 995-1001. 10.1139/f89-129

[JEB250437C80] Wanzenböck, J., Zaunreiter, M., Wahl, C. M. and Noakes, D. L. G. (1996). Comparison of behavioural and morphological measures of visual resolution during ontogeny of roach (*Rutilus rutilus*) and yellow perch (*Perca flavescens*). *Can. J. Fish. Aquat. Sci.* 53, 1506-1512. 10.1139/cjfas-53-7-1506

[JEB250437C81] Warrant, E. J. and Nilsson, D. E. (1998). Absorption of white light in photoreceptors. *Vision Res.* 38, 195-207. 10.1016/S0042-6989(97)00151-X 9536349

[JEB250437C82] Watanuki, N., Kawamura, G., Kaneuchi, S. and Iwashita, T. (2000). Role of vision in behavior, visual field, and visual acuity of cuttlefish *Sepia esculenta*. *Fish. Sci.* 66, 417-423. 10.1046/j.1444-2906.2000.00068.x

[JEB250437C83] West, J. A., Sivak, J. G. and Doughty, M. J. (1995). Microscopical evaluation of the crystalline lens of the squid (*Loligo opalescens*) during embryonic development. *Exp. Eye Res.* 60, 19-35. 10.1016/S0014-4835(05)80080-6 7720802

[JEB250437C84] Yamamoto, T., Tasaki, K., Sugawara, Y. and Tonosaki, A. (1965). Fine structure of the octopus retina. *J. Cell Biol.* 25, 345-359. 10.1083/jcb.25.2.345 14287185 PMC2106631

[JEB250437C85] Young, J. Z. (1962). The retina of cephalopods and its degeneration after optic nerve section. *Philos. Trans. R. Soc. Lond. B. Biol. Sci.* 245, 1-18. 10.1098/rstb.1962.0004

[JEB250437C86] Young, J. Z. and Harman, R. F. (1988). “Larva”, “paralarva” and “subadult” in cephalopod terminology. *Malacologia* 29, 201-207.

